# Hand rearing affects emotional responses but not basic cognitive performance in European starlings^☆^

**DOI:** 10.1016/j.anbehav.2013.05.002

**Published:** 2013-07

**Authors:** Gesa Feenders, Melissa Bateson

**Affiliations:** Centre for Behaviour and Evolution, Institute of Neuroscience, Newcastle University, Newcastle upon Tyne, U.K.

**Keywords:** animal welfare, behavioural disinhibition, early life stress, European starling, impulsivity, maternal deprivation, perseveration, *Sturnus vulgaris*

## Abstract

Hand rearing is a common procedure in behavioural research on birds. While likely to produce tamer experimental animals, there is a risk that it could induce pathological changes in brain and behaviour similar to those seen in mammals that have experienced maternal separation. We explored the effects of hand rearing on the cognitive and behavioural development of European starlings, *Sturnus vulgaris*, to assess the generality of results obtained from hand-reared animals. Two groups of age-matched birds were created from the same wild population: one hand-reared from 10 days posthatch and one brought into the laboratory as independent juveniles. These groups were compared on a battery of neuropsychological tasks designed to probe different aspects of cognitive function including learning, perseverative cognition, interval timing, neophobia and impulsivity. There was no evidence for cognitive impairment in the hand-reared birds. They did not have reduced learning speed, impairments in accuracy or precision of interval timing or pathological perseverative cognition compared to the wild-caught birds. Additionally, there was no evidence that birds that developed stereotypies in laboratory cages (predominantly the wild-caught birds) had any cognitive impairments, although this may be because no birds had severe, crystallized stereotypies. There was some evidence that hand-reared birds were less neophobic and less impulsive than wild-caught birds, suggesting that hand rearing might alter emotionally mediated decision making in a direction usually associated with reduced developmental stress in mammals. This study therefore supports the use of hand rearing as an experimental procedure in behavioural research on passerine birds.

## Highlights

• We used neuropsychological tests to study the effects of hand rearing in starlings.
• Hand rearing did not affect associative learning, interval timing or perseveration.
• Cognitive perseveration was not correlated with stereotypic behaviour.
• Hand-reared birds were less fearful and also less impulsive.
• Hand rearing alters emotional responses but not basic cognition.

Hand rearing experimental animals is a common procedure in behavioural research. Reasons for hand rearing include control of maternal effects (e.g. Madruga et al. 2006; Hulshof et al. 2011), manipulation of experience or diet during development (Thorpe 1958; Nowicki et al. 2002; Exnerova et al. 2006) and, via extensive human handling, habituation of animals to humans and laboratory procedures (e.g. Jones & Waddington 1992; Bilko & Altbacker 2000). The latter motivation for hand rearing is particularly prevalent in recent studies of nondomesticated passerine bird species that rely on well-habituated experimental subjects (e.g. Clayton & Dickinson 1998; Seed et al. 2007; Hoffmann et al. 2011; Schwab et al. 2012). In support of the practice, there is evidence that early handling reduces fear of humans and the stress reaction to restraint in parrots (Aengus & Millam 1999; Collette et al. 2000), and recent experimental studies have confirmed that hand-reared starlings are less fearful of humans and novel environments compared to birds caught from the wild as adults (Feenders & Bateson 2011; Feenders et al. 2011; Jayne et al. 2013). While hand rearing is undoubtedly a valuable experimental tool, there is reason to believe that it could have profound effects on the cognition and behaviour of adult animals, some of which are potentially indicative of pathological changes in the brain. Therefore, to assess the generality of scientific findings from hand-reared birds, it is important to understand how hand rearing affects behavioural development, and specifically whether the cognitive and behavioural phenotypes of adult hand-reared birds are abnormal. We addressed this question by conducting a neuropsychological investigation of the effects of hand rearing in the European starling, *Sturnus vulgaris*, the wild passerine most commonly used in laboratory research (Bateson & Feenders 2010).

Hand rearing usually involves removal of the young animals from their parents, and often also from the wild, shortly after birth or hatching. The young animals are subsequently housed in the laboratory, either in isolation or in peer groups, and are reared by human care-givers using replacement diets (e.g. Feenders & Bateson 2011). Hand rearing therefore alters several aspects of early life experience, including the quantity and quality of maternal care, the physical and social environment and the developmental diet.

In humans, poor parenting and adverse experiences during early development are associated with impairments in adult cognitive ability and an increased risk for developing psychiatric disorders such as anxiety, depression and psychoses (Kaufman et al. 2000; Heim & Nemeroff 2001; McEwen 2003). There have been numerous attempts to model these effects in nonhuman animals. In mammals, many studies have explored how manipulating specific aspects of maternal care shapes the adult physiological, neurobiological and behavioural phenotype. In rats, *Rattus norvegicus*, maternal separation produces long-lasting changes in emotional behaviour and impaired responses to stress (Anisman et al. 1998; Meaney 2001; Pryce & Feldon 2003; Macrì & Würbel 2006). Maternal separation also induces reduced neurogenesis in the adult hippocampus and consequential impairments in learning and memory (Korosi et al. 2012). In rhesus monkeys, *Macaca mulatta*, removal from the mother followed by peer rearing or rearing by mothers experiencing variable foraging conditions produces adults with more reactive stress physiology, increased anxiety, impulsivity and aggression and behavioural abnormalities such as motor stereotypies (e.g. Hennessy 1997; Cirulli et al. 2009; Nelson et al. 2009; Stevens et al. 2009; Prescott et al. 2012). Furthermore, adverse events during early development have been shown to increase the likelihood of developing abnormal behaviour, and specifically motor stereotypies, in a range of species. For example, animals removed from their mother at an earlier age, and animals born in captive as opposed to natural environments, show a higher incidence of stereotypic behaviour (Mason 2006; Latham & Mason 2008; Jones et al. 2011). Stereotypies are of concern because they are associated with executive dysfunction involving inappropriate repetition of responses (perseveration) and pathological changes in the underlying basal ganglia circuitry (Sandson & Albert 1984; Garner 2006; Langen et al. 2011).

In birds, there is some evidence that manipulations that involve elements of hand rearing affect the adult phenotypes similarly to the effects observed in mammals. For example, zebra finches, *Taeniopygia guttata*, fed corticosterone as chicks exhibit exaggerated and prolonged responses to acute stress as adults (Spencer et al. 2009), and in various passerine models reducing the quantity or quality of food fed to chicks impairs learning (Nowicki et al. 2002; Fisher et al. 2006). The picture in relation to stereotypies is less clear. In parrots, like mammals, early maternal separation leads to an increase in stereotypic behaviour (Schmid et al. 2006), and in blue jays, *Cyanocitta cristata*, hand-reared birds perform more spot-pecking stereotypies but less route tracing than wild-caught conspecifics (Keiper 1969). In contrast, hand-reared European starlings exhibit higher activity but less stereotypic route tracing and somersaulting compared with wild-caught conspecifics (Feenders & Bateson 2012). There is some evidence that motor stereotypies are associated with perseverative cognition indicative of basal ganglia pathology in both parrots and passerines (Garner et al. 2003a, b).

In summary, there is a body of evidence suggesting that manipulations that include one or more elements of the hand-rearing procedure result in adult animals with more reactive stress physiology, impaired cognitive performance, increased anxiety and impulsivity and a higher incidence of abnormal stereotypic behaviour. However, there have been few studies of the effects of hand rearing per se in birds. Our aim in this study was to establish the impact of hand rearing on cognitive performance in European starlings. We created two groups of age-matched birds from the same wild population: a hand-reared group brought into the laboratory 10 days posthatch and reared in peer groups by humans and a wild-caught group reared by their parents in the wild and subsequently caught and brought into the laboratory as independent juveniles. We used a battery of established neuropsychological tests to compare the two groups. In addition, we quantified the incidence of stereotypic behaviour in both groups of birds to establish whether behavioural stereotypies (in this case route tracing and stereotypic somersaulting) are associated with perseverative cognition or other cognitive impairments. Below we briefly describe the neuropsychological tests that we chose and outline our predictions.

### Autoshaping

Autoshaping can be used to measure the speed of associative learning. During an autoshaping procedure, a novel conditioned stimulus (CS, in this case a key light) predicts unconditional delivery of a food reward (US; e.g. Bateson & Kacelnik 1995). The learning of an association between the CS and US is evidenced by appetitive responses (pecking) directed at the CS. We predicted that hand-reared birds would show impaired speed of learning due to early maternal separation or deficits in early nutrition.

### Novel Stimulus

Response to a novel object or stimulus is a measure of neophobia and anxiety (e.g. Meehan & Mench 2002; Drent et al. 2003; Forkman et al. 2007; Feenders et al. 2011). In this study the task was implemented by changing the colour of the stimulus (key light) used for autoshaping. We predicted that if hand-reared birds are more anxious as a result of early stress, they would be more neophobic and slower to peck the novel colour.

### Extinction

Extinction of a previously learnt association is a measure of cognitive perseveration. During extinction learning, the subject is first rewarded for responding to a CS, for example pecking an illuminated key; then, the reward is ceased and persistence in responding is measured. Subjects with cognitive perseveration tend to persist longer than normal subjects (Garner et al. 2003a). We predicted higher levels of perseveration in birds performing more stereotypic behaviour.

### Two-choice Guessing Task

The two-choice guessing (or gambling) task is a standard test of cognitive perseveration. In this task, the subject is presented with a choice of two operanda and challenged to find the ‘correct’ (i.e. rewarded) option. In reality, the reward is randomly assigned to the two options meaning that there is no correct response. Normal subjects will explore both options, randomly alternating between them, whereas perseverative subjects persist in responding on one alternative (e.g. continue to press the left key) for extended periods (Frith & Done 1983; Garner 2006). As for extinction, we predicted higher levels of perseveration in those birds performing higher levels of stereotypic behaviour.

### Sensitivity to Risk in Delay to Reward

Decision making when a subject is presented with a choice between a fixed delay to reward versus a risky delay (that is either shorter or longer with equal probability) provides a measure of impulsivity (Bateson & Kacelnik 1996, 1997; Kacelnik & Bateson 1997). When given a choice between a fixed delay to reward and a risky delay with the same arithmetic mean, animals universally prefer the risky option (Kacelnik & Bateson 1996). This preference occurs because animals discount delayed rewards hyperbolically with time; the risky option has a higher value due to the short delays to reward it contains. The fixed delay can be titrated until the subject is indifferent to obtain a quantitative estimate of how rapidly delayed rewards are discounted, with smaller values of the fixed delay at indifference indicating steeper time discounting and hence greater impulsivity (Bateson & Kacelnik 1996, 1997). Thus, if hand-reared birds are more impulsive, then we predicted that they should have smaller values of the fixed delay at indifference.

This task has the added bonus of allowing us to measure interval-timing performance, which is a good indicator of various types of neuropathology, including pathologies of the basal ganglia circuits (Paule et al. 1999; Matell & Meck 2004). During the long delays to reward, the bird's pecking distribution can be recorded to extract timing accuracy (peak time of the pecking distribution with respect to the actual timing of reward), timing precision (width of the pecking distribution) and motivation (peak rate of pecking). Since the basal ganglia system is implicated in interval timing, we predicted stereotyping starlings would show altered timing functions as a result of the basal ganglia dysfunction: perseverative pecking would lead to timing functions that were broader and shifted to longer intervals.

## Methods

### Subjects and Housing Conditions

Thirty-two starlings were used, 16 hand-reared (HR; nine females and seven males) and 16 wild-caught (WC; nine females and seven males). All birds originated from the same population of starlings in northeast England. They were hatched in 2009 and were of a similar age (starlings raise a single yearly brood in the north of England, and broods hatch approximately synchronously). We took steps to ensure that the 32 birds were not closely related (i.e. not siblings). The HR group was taken from 16 different nestboxes as nestlings (ca. 10 days old). Groups of four or five chicks were placed in artificial nests comprising plastic boxes (20 × 20 cm and 10 cm high) lined with paper towels and loosely covered with thick paper. The chicks were transported to the laboratory by car for hand rearing. The chicks were initially fed to satiation approximately every 30 min for 14 h per day on a mix of soaked dry cat food and apple sauce, supplemented with vitamins (BSP drops, Vetark) and calcium (Zolcal D, Vetark). The frequency of feeds was gradually reduced as the birds grew (for further details of husbandry procedures see Feenders & Bateson 2011). Once they became independent (ca. 4 weeks posthatch), they were transferred to an indoor aviary (215 × 340 cm and 220 cm high; ca. 19 °C; 14:10 h light:dark), provided with environmental enrichment consisting of bark chips on the floor as a natural foraging substrate, a water bath, and ropes for perching. The birds were fed ad libitum on domestic chick crumbs supplemented with dried insect food (Orlux), mealworms and fruit. The WC group was caught in the autumn of the same year (when juveniles could still be clearly identified from their plumage) with a baited whoosh net. Birds were removed from the net and placed into individual cloth bags for the car journey to the laboratory where they were housed in a separate indoor aviary under identical housing conditions to the HR group. We know that the WC birds originated from different clutches from the HR birds because the remaining chicks from clutches used for taking the HR nestlings were ringed for subsequent identification and the WC birds were taken from a flock of several hundred birds making it unlikely that any two WC birds came from the same clutch.

Tarsus length was measured once during the study after skeletal growth was complete. Body weights were taken every time the birds were moved in or out of the experimental cages. Residuals from the regression of body weight on tarsus length were calculated for each of those four time points as a measure of body condition.

Our study was approved by Newcastle University's Ethical Review Committee. The starlings were taken from the wild under Natural England licence 20093194. After the experiment the birds were retained for further studies.

### Experimental Cages

For the cognitive tests the starlings were transferred to individual experimental cages that served both for cognitive testing and as their home cages during the periods of testing. The cages measured 100 × 45 cm and 45 cm high and had wire-mesh front and back walls, solid side walls and floor and a transparent Perspex roof. The temperature and lighting conditions were the same in the experimental room as in the aviary (ca. 19 °C; 14:10 h light:dark); thus for the entire experiment the birds were maintained on long days and hence in nonreproductive condition. Eight cages were arranged in the experimental room on two rows of shelves (at 38 and 120 cm from the floor) such that the birds could hear each other and each bird could see four to six other birds. The cages were identical with the exception that four were environmentally enriched with a small hide, a tray of sawdust for foraging and a water bath, while the other four had no hide and an empty foraging tray and bath (the bath was filled twice a week for 1 h to allow basic hygiene). Previous data from this group of birds (Feenders & Bateson 2011; Feenders et al. 2011) and preliminary analyses of the current data set did not reveal any effect of current housing condition (enriched versus nonenriched); thus, we decided to exclude housing as a between-subjects factor in the current experiment to maximize power.

**Figure 1:**
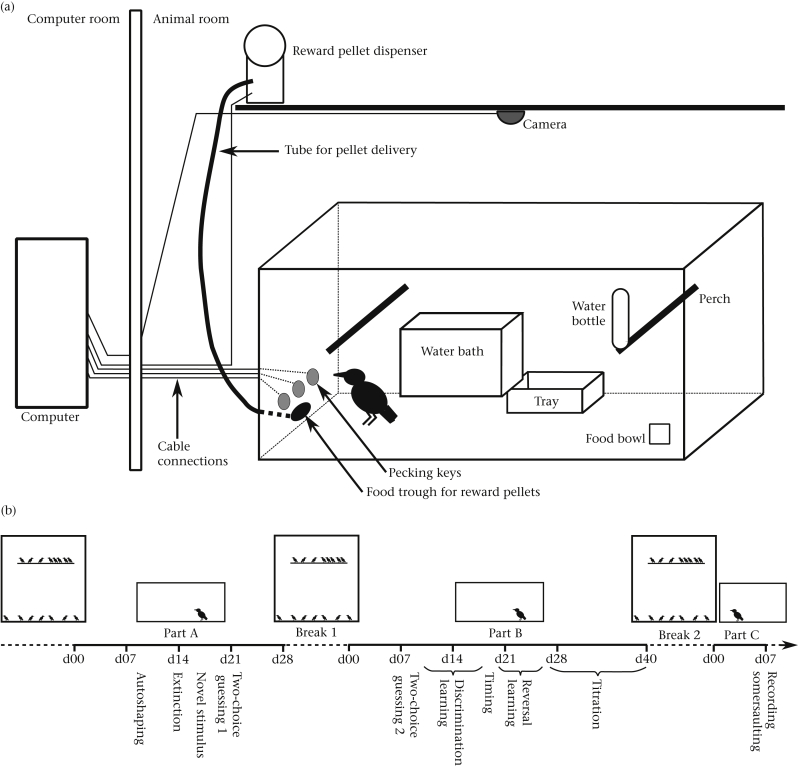
(a) Experimental set-up to test starlings in operant tasks. The home cage is equipped with three remotely controlled pecking keys and a food dispenser for reward pellets. The bird's behaviour can be recorded by a video camera mounted above the cage. (b) Schematic of the experimental schedule showing the change between aviaries (large squares with multiple birds) and individual cages (smaller squares with one bird) along the three parts of the study. Horizontal line marks the timeline, with periods of varying lengths in aviaries indicated by dashed lines, periods of mostly fixed length in test cages marked by solid lines and timing of the different tests presented in this study. Birds in cages/aviaries are drawn approximately to scale; aviaries are not in scale to cages. Cage/aviary furnishing is not shown.

Each cage was fitted with an overhead surveillance camera (Atom, CSP Technology, Scunthorpe, U.K.) connected to an adjacent room for remote observation and recording. A custom-built operant panel comprising three horizontally aligned 4 cm diameter pigeon pecking keys and one central food trough was permanently attached to one of the side walls of each cage (components from Campden Instruments, Loughborough, U.K.). The two outer keys could be transilluminated with either green or red light, and the centre key with amber light. The trough was connected to an external pellet dispenser (Campden Instruments, Loughborough, U.K.) delivering 45 mg, grain-based rodent pellets (TestDiet, Richmond, IN, U.S.A.). The food dispensers as well as the pecking keys were connected via interfacing hardware (CeNeS, Cambridge, U.K.) to a computer running the Whisker Experimental Control system (Cardinal & Aitken 2010), which controlled stimulus events and response contingencies as well as recording the data (Fig. 1a). The cognitive tasks used in this study were custom-written in Microsoft Visual Basic 5.0 (Microsoft Corporation, Redmond, WA, U.S.A.).

### Cognitive Testing

#### General procedures

The study was divided into two main parts. In part A, we sequentially tested four replicate groups of eight birds, each group consisting of four HR and four WC birds. The birds were assigned to the cages in a counterbalanced fashion with respect to developmental history (HR versus WC) and enrichment condition (enriched versus nonenriched). Part A (ca. 30 days per replicate) took place in February–June 2010 when birds were approximately 1 year old. In part B, 16 birds (eight HR, eight WC) were tested in two sequential replicate groups (ca. 36 days per replicate) in July–October of the same year. In part B, all cages were environmentally enriched. During operant training and testing in both part A and part B, the birds were food deprived overnight from 1700 hours until testing began the following morning at 0800 hours in order to motivate them to work for food. Since the lights were off from 2100 to 0700 hours, and starlings do not eat during the night, they experienced a total of 5 h of additional food deprivation on top of the night hours. This period of food deprivation is within the normal daily range experienced by wild starlings; local starlings experience nights of in excess of 15 h for 3 months each year, and a maximum night length of nearly 17 h at the winter solstice. Therefore these birds are adapted for coping with prolonged periods without eating on a daily basis, usually coupled with much lower temperatures than those in our laboratory. Operant sessions lasted for a maximum of 4 h per day and at 1200 hours each day general husbandry was performed on the cages and the birds were given ad libitum food until 1700 hours. The birds were weighed when they were first put into the experimental cages and periodically during testing. The above regime resulted in the birds maintaining stable body weights approximately 5% below their free-feeding weights (birds lost an average of 5.78% of their starting weight in part A and 4.55% in part B). When not being tested between parts A and B, the HR and the WC birds were returned to their respective aviaries (described above). Figure 1b illustrates the schedule of the tests.

#### Autoshaping and operant training (part A)

Initially, the birds were autoshaped to peck the centre amber key for a food reward: the centre key was lit for 40 s (unless the bird pecked earlier, in which case the light was extinguished immediately), followed by delivery of two pellets (1/s) and an intertrial interval (ITI) of 400 s. A total of 33 such trials were given per day. If within 2 days a bird had not started to peck the key (10 birds), the bird was confined to a space close to the operant panel by placing a partition into the cage prior to the session. This procedure made nine birds initiate pecking on the third day, but one bird only started pecking after a mealworm had been taped to the pecking key (after completion of the morning session). Another bird had to be tutored to feed on the reward pellets during the autoshaping procedure. All 32 birds finally learnt to peck the amber key for reward. Once a bird started to peck the key, the next day it progressed to a variable number of days of operant training. For operant training, the stimulus time was reduced to 15 s (as before, once a peck occurred the light was extinguished immediately), the food reward was conditional on a peck and the ITI was reduced to 200 s. Each bird received daily sessions of 60 such trials until it had pecked on at least 80% of trials in three sessions. When a bird had met this criterion it progressed to the cognitive test series starting on the following day with extinction learning (see next section).

The number of trials until the first peck occurred was recorded as a measure of speed of associative learning. The bird that only started to peck after a worm had been taped to the key was assigned a value of 100 trials (as it did not peck during the first 99 trials); the bird that had to be tutored was excluded from this data set as it did not follow the same training procedure as the other birds. The average latency to peck from the three 60-trial sessions with a peck rate of ≥80% per day was computed as a measure of motivation to peck during the final stage of training.

#### Cognitive tasks

In the following tasks, stimulus times are given as the maximum duration of key illumination possible; if the bird pecked earlier, the light was extinguished immediately and the trial progressed. To ensure that birds had a similar level of pecking performance prior to each task, a bird only proceeded directly from one task to the next if it had pecked in ≥80% of the trials (extinction learning recovery, novel stimulus task, forced choice task; see below); otherwise the task was repeated until criterion (pecks in ≥80% of trials). The food reward consisted of two pellets throughout part A and one pellet throughout part B.

#### Extinction learning (part A)

Each bird received a single session comprising 10 rewarded trials (centre amber light 15 s, ITI 150 s) followed by 60 unrewarded trials (same stimulus colour and duration). The number of trials on which a bird pecked during the final 10 trials of this session was recorded as a measure of extinction learning. On the next day the bird received a recovery session (60 trials) with the previously unrewarded stimulus rewarded again.

#### Novel stimulus (part A)

In this test the stimulus was changed from an amber light on the centre key to a green light on either the right or left key (side counterbalanced for origin, enrichment and cage position) for 15 s, followed by 200 s ITI. A total of 60 trials were presented. The number of trials until the first peck occurred was recorded as a measure of willingness to generalize to a new stimulus colour/position. One wild-caught bird did not peck during the session and was assigned a maximum value of 61 trials. This test was repeated, but now with the stimulus being changed to red. All birds except one started to peck on trials 1–3 (25 of 32 birds on trial 1), so there was not enough variation in the birds' performance to analyse.

#### Forced choice (part A)

The aim of this task was to train the birds to peck all three keys and to attend to the test stimulus (i.e. a trial without a peck was not due to averted attention). For this, birds were presented with an initiation stimulus to start the actual test stimulus: once the centre amber light was illuminated (no time limit), a peck to this key resulted in one of the outer keys being illuminated in red for 15 s; a peck to this test stimulus was rewarded, followed by 200 s ITI. The test comprised 60 trials and the test stimulus was pseudorandomly assigned to the two outer keys as follows. After each block of 20 trials, the side bias was adjusted for the following 10 trials: for example if the test stimulus had appeared in 14 trials on the left and in six trials on the right, in the following eight (i.e. 14–6) trials the stimulus would appear on the right key to equalize the overall number of trials presented on the right and left. As this task was merely for training purposes and all birds readily followed the rule, no behavioural measures were extracted.

#### Two-choice guessing task (part A and B)

This task was performed in two different versions: a version without side bias correction was used in part A, whereas a version with side bias correction was used in part B. In part A, a trial started with the centre amber key being illuminated (no time limit). When the bird pecked this key it was extinguished and the two outer keys were illuminated in red for 15 s. When the bird pecked one of these keys (i.e. made a choice) both keys were extinguished; the reward was randomly assigned to one of the two outer keys, thus a choice was rewarded with a probability of 0.5. If the bird did not peck within 15 s stimulus-on time, the trial was terminated and the ITI started; this occurred very rarely (a maximum of three trials per day in a few birds). The ITI was set to 150 s and a total of 80 trials were presented. This task was repeated over 3 consecutive days. After completion of the task, the side bias was extracted separately for each day, expressed as the number of trials to the preferred side in proportion to the total number of trials completed. One bird stopped pecking on day 1, and another bird stopped pecking on day 2 and day 3; these incomplete days were excluded from the analysis. Owing to a very strong side bias in almost all birds, no other metric could be extracted from this task (see Discussion).

In part B, a side bias-corrected version of the task was used (Garner et al. 2003b): the assignment of the reward was based on the choice probability of the bird in the preceding 20 trials. For example, if a bird chose the left key in eight of the 20 trials, the probability of the reward to be assigned to the left key in the current trial was 0.6 (1 − (8/20)). The test comprised two blocks of 150 trials each, separated by a 30 min break to prevent satiation. After a bird pecked the initiation stimulus (the centre amber key, no time-out), both outer keys were illuminated green for a maximum of 15 s, followed by 30 s ITI. The bird's choice was recorded. If a bird did not peck within the 15 s stimulus-on time (two trials in one bird), this trial was assigned the choice of the preceding trial. Then, the sequence of left–right choices was analysed using a third-order Markov-chain approach to get a sequential dependency score (Garner et al. 2003a): the total sequence of 300 trials was split into six subsequences of 50 trials length, and for each subsequence the expected probability of each combination of four consecutive choices (tetragrams) was calculated based on the actual side bias of each subsequence. This expected probability was compared with the actual occurring probability using a chi-square analysis, resulting in a chi-square sum and related *P* value. Here, a *P* value close to 1 indicates randomness (sequential independence), while a *P* value close to 0 indicates high sequential dependency. Finally, the averages of the chi-square sums from each subsequence were calculated to obtain one average score of sequential dependency per bird.

#### Impulsivity and interval timing (part B)

In the risk sensitivity task, the bird's valuation of a delayed reward was assessed by measuring the preference for a variable delay (short or long) over a fixed delay (intermediate) using a titration procedure. Birds were presented with two different stimuli (red and green key lights, assignment counterbalanced for origin and cage position): the reward was either delivered after a fixed delay of initially 10 s (henceforth FD), or after a variable delay of either 3 or 15 s with 50% probability (henceforth VD). Each trial started with the initiation stimulus (centre amber key) to ensure the bird was attending to the operant panel. When the initiation stimulus was pecked, one (no-choice trial) or two (choice trial) test stimuli started to flash (0.3 s on, 0.7 s off; always outer keys); as soon as the bird pecked, the chosen light was turned on continuously, while the other stimulus was turned off (only applicable during choice trials) and the stimulus-related delay (fixed/variable) started. During the delay, the number of pecks was recorded in 1 s time bins. The first peck that occurred after the scheduled delay had passed resulted in delivery of the food reward, followed by a 40 s ITI. Trials were arranged in blocks of four, with the first two trials being no-choice trials, one each with the FD and the VD stimulus (side and order randomized), followed by two choice trials in which both stimuli were presented for the bird to choose between (sides randomized). Each bird was tested for two sessions of 25 blocks, with a 30 min break between the sessions to avoid satiation. Testing was stopped when the total of 50 blocks (200 trials in total) was completed or 4 h had elapsed, whichever came first. The FD was adjusted each session according to the following titration protocol (%VD indicating the percentage of choices made to the VD stimulus in the choice trials).

(1) Step 1: FD was started at 10 s (expectation: clear preference for VD). The bird was trained until either %VD ≥ 90% for 4 consecutive days or 15 days of training had been completed and the %VD was significant for the last 6 days (i.e. %VD > 50% every day for 6 consecutive days) to ensure that the bird had properly learned the task.

(2) Step 2: when the above criterion was met, FD was changed to 3 s (expectation: clear preference for FD). The bird was trained until either %VD choice ≤ 10% on 1 day or %VD < 50% on 6 consecutive days.

(3) Step 3: when the above criterion was met, FD was changed to 6.5 s (since this is halfway between 3 and 10 s; expectation: some preference for VD as this value is higher than the harmonic mean). The bird was trained until %VD > 50% for 1 day.

(4) Step 4: when the above criterion was met, FD was changed to 4.8 s (halfway between 3 and 6.5). At this point FD was titrated each day according to the following rules: if %VD > 50% → FD = FD − 0.2 s; if %VD = 50% → FD unchanged; if %VD < 50% → FD = FD + 0.2 s.

For the analysis, the number of trials the bird completed to reach the criterion in step 1 (clear preference for VD) was extracted as a measure of learning performance, and the number of trials completed to reach the criterion of step 2 was used as an indicator of reversal learning (here, the bird had to change preference from VD to FD). The actual VD preference on the last day of step 1 indicated the strength of preference. The average value of the fixed delay during the first 5 days of the titration process was taken as an indicator of risk sensitivity (one WC bird completed only 4 days of titration, in which case the average was based on those 4 days).

**Figure 2:**
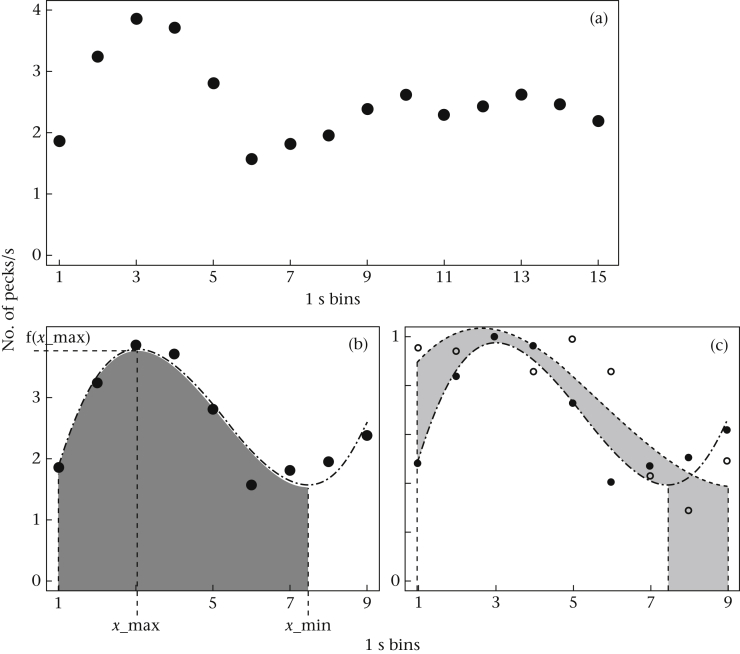
Analysis of the timing curve. (a) Example of a pecking distribution (expressed as number of pecks per 1 s time bin) across a 15 s delay; average of 21 trials of one individual. (b) Extracting timing parameters by fitting a third-order polynomial function (dash-dotted line) to the pecking distribution (filled circles, as in (a)) including time bins 1–9 s: *x*-max is the *x* value of the maximum, indicating accuracy; f(*x*_max) is the corresponding *y* value indicating motivation; the grey shaded area under the curve indicates timing precision (extracted from a normalized curve as shown in (c)). (c) Normalized curve “maximum pecking rate = 1” of the same bird as shown in (a, b) (filled circles, dash-dotted line) compared to a bird with lower timing precision (open circles, dashed line; average of 23 trials); the difference in precision (area under the curve) is marked in light grey shading.

To analyse interval timing performance, we used the last day of FD = 10 s (i.e. prior to proceeding to step 2) to examine the average pecking frequencies during the no-choice trials with long delays (VD = 15 s). During these trials the birds typically showed a peak of pecking at around 3 s (the position of the short-delay reward), followed by a decrease until ca. 8 s (a delay without associated reward) and a final increase towards the 15 s delay (Fig. 2a). Therefore, these data can be used to measure the birds' performance at reproducing a 3 s time interval. To do this, we fitted the first 9 s of data with a third-order polynomial function. From the fitted functions, the *x* value of the first maximum was extracted as a measure of timing accuracy for the short delay and the *y* value of the first maximum as a measure of motivation (Fig. 2b). To obtain a measure of timing precision, a third-order polynomial function was fitted to the pecking distribution now normalized to the maximum pecking rate (i.e. the pecking motivation; the value of the time bin where the maximum number of pecks occurred was set to 1 and the number of pecks in the other time bins adjusted accordingly) and the area under the curve was calculated from time 0 s to the *x* value of the function's minimum.

#### General activity and route-tracing stereotypies

The birds' behaviour in the experimental cages was recorded every other day for 1 h in the morning starting at 0700 hours (when lights went on). The videos were analysed using the tracking software EthoVision XT v5.1 (Noldus Information Technology, Wageningen, The Netherlands; for details, see Feenders & Bateson 2012). The bird's general activity level (*T*_move_) was measured as the total length of time it spent moving (>10 cm/s). Use of the cage walls (*F*_walls_, previously discussed as an indicator of escape attempts by Maddocks et al. 2002) was measured as the total number of visits to the walls as a proportion of the total number of location changes.

The sequence of position changes (coded as distinct cage locations) was transferred from the EthoVision into the Theme software package (Noldus Information Technology) to measure recurring sequences of events (T patterns) as a measure of route tracing (for a detailed discussion of Theme and this approach see Brilot et al. 2009; Feenders & Bateson 2012). From this analysis, the number of different T patterns (PatDiff) and the total number of T-pattern occurrences (PatOcc) were extracted as measures of route-tracing behaviour.

In analyses of correlations between behaviour and cognition we used the average of a behavioural variable (T_move_, F_walls_, PatDiff, PatOcc) from the 2 days nearest to the day on which the relevant cognitive data were collected.

#### Somersaulting stereotypies

We intended to use somersaulting behaviour, the most prominent stereotypy found in starlings, as a measure of stereotypy. However, as we observed almost no somersaulting in our experimental cages we therefore additionally tested all the birds in standard cages known to elicit this behaviour in at least some proportion (10–40%) of birds (Asher et al. 2009; Brilot et al. 2009, 2010). Birds were categorized as either ‘somersaulting’ (SOM) or ‘normal’ (NML) as described in Feenders & Bateson (2012).

### Data Analysis

Most data did not meet assumptions of normality even after transformation. Therefore, we used nonparametric tests throughout: Mann–Whitney tests for comparison of independent samples and Spearman rank for correlations. All statistical analyses were performed in SPSS version 19 (SPSS Inc., Chicago, IL, U.S.A.). We adopted an alpha value of 0.05 and used two-tailed tests throughout. For Mann–Whitney tests we report exact *P* values because sample sizes did not always meet the threshold values for asymptotic testing (Mundry & Fischer 1998).

## Results

Throughout we use the following notation to indicate sample sizes of the different groups: *N*_wc_ = sample size of wild-caught group, *N*_hr_ = hand-reared, *N*_nml_ = wild-caught normal, *N*_som_ = wild-caught somersaulting, *N*_total_ = sample size for correlations including HR and WC. Since stereotypic somersaulting behaviours only occurred in the wild-caught group (*N*_nml_ = 10 and *N*_som_ = 6), comparisons of the cognitive performance in normal and somersaulting birds are only made for the wild-caught birds. Correlates of somersaulting were only examined in part A because the sample size in part B was too small.

### Body Condition

Body condition was significantly correlated at the beginning and end of parts A (*r*_S_ = 0.674, *N*_total_ = 32, *P* < 0.001) and B (*r*_S_ = 0.759, *N*_total_ = 16, *P* = 0.001) of the study, suggesting consistent individual differences in body condition. Body condition was not affected by origin at any time point in part A (*U* ≤ 152, *N*_wc_ = *N*_hr_ = 16, *P* ≥ 0.381) or part B (*U* ≤ 42, *N*_wc_ = *N*_hr_ = 8, *P* ≥ 0.328). Body condition at the end of part A of the study had no effect on whether starlings developed somersaulting behaviour (*U* = 35, *N*_nml_ = 10, *N*_som_ = 6, *P* = 0.635).

### Autoshaping

**Figure 3:**
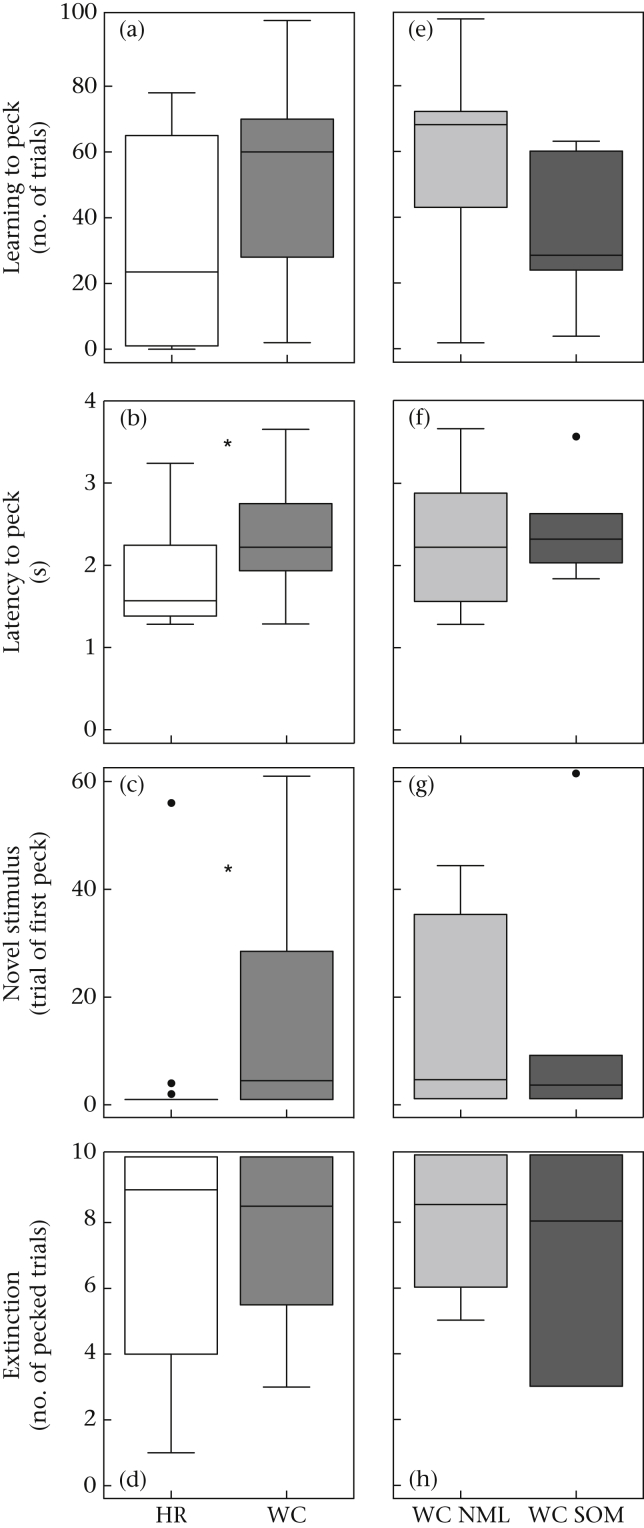
Performance during tasks in part A. (a, e) Number of trials to start to peck during autoshaping. (b, f) latency to peck during training trials, (c, g) number of trials to peck a novel stimulus, (d, h) number of trials to extinction. Asterisks indicate significant difference (*P* ≤ 0.05). Box plots indicate median with first and third quartiles (box), 1.5 interquartile ranges (whiskers) and outliers (dots). (a–d) HR (white boxes): hand-reared; WC (grey boxes): wild-caught; (e–h) WC NML (light-grey boxes): WC not showing somersaulting; WC SOM (dark-grey boxes): WC showing somersaulting.

Hand-reared birds took fewer trials than wild-caught birds to start to peck the key (near-significant trend: *U* = 163.5, *N*_wc_ = 15, *N*_hr_ = 16, *P* = 0.086; Fig. 3a). They also had shorter latencies to peck during early training (*U* = 182, *N*_wc_ = *N*_hr_ = 16, *P* = 0.043; Fig. 3b). Within the wild-caught birds, somersaulting birds took fewer trials to start pecking (near-significant trend: *U* = 11, *N*_nml_ = 9, *N*_som_ = 6, *P* = 0.066; Fig. 3e), but latency to peck was not affected by somersaulting status (*U* = 32, *N*_nml_ = 10, *N*_som_ = 6, *P* = 0.875; Fig. 3f).

### Novel Stimulus

Hand-reared birds took fewer trials to start pecking at a novel stimulus than wild-caught birds (*U* = 188, *N*_wc_ = *N*_hr_ = 16, *P* = 0.023; Fig. 3c). Within the wild-caught birds, performance on this task was not affected by somersaulting status (*U* = 30.5, *N*_nml_ = 10, *N*_som_ = 6, *P* > 0.999; Fig. 3g). Across all birds, performance in this task was weakly correlated with trials to start pecking during autoshaping (*r*_S_ = 0.323, *N*_total_ = 31, *P* = 0.076) suggesting consistent individual differences in reluctance to approach and peck an illuminated key.

### Extinction

Extinction learning was not affected by origin (*U* = 134.5, *N*_wc_ = *N*_hr_ = 16, *P* = 0.809; Fig. 3d) or somersaulting status (*U* = 26.5, *N*_nml_ = 10, *N*_som_ = 6, *P* = 0.713; Fig. 3h). Across all birds, perseveration on this task was correlated with general activity (*r*_S_ = 0.440, *N*_total_ = 32, *P* = 0.012), but was not related to use of cage walls or either route-tracing metric (*r*_S_ < 0.232, *N*_total_ = 32, *P* ≥ 0.294 in all cases).

### Two-choice Guessing Task

**Figure 4:**
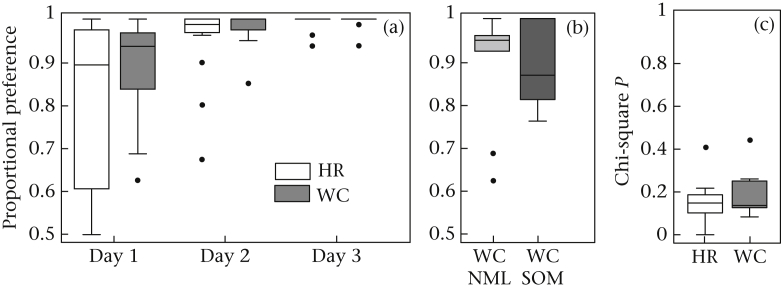
Performance during the two-choice guessing task. (a) Side bias during the 3 consecutive days of testing in part A, (b) side bias on the first day of part A, (c) randomness, as measured in the task during part B. Box plots as explained in legend of Fig. 3. HR: hand-reared; WC: wild-caught; WC NML: WC not showing somersaulting; WC SOM: WC showing somersaulting.

In part A, performance on the two-choice guessing task was mainly driven by side biases. The degree of side bias (irrespective of what side was preferred) was not affected by origin (*U* = 92.5, *N*_wc_ = 15, *N*_hr_ = 16, *P* = 0.285; Fig. 4a) or somersaulting status (*U* = 29, *N*_nml_ = 9, *N*_som_ = 6, *P* = 0.859; Fig. 4b). The strong side biases in this experiment invalidated any analysis of the response sequences.

In part B, side biases were very weak (mean + SEM = 56.9 + 2.24%), showing that the revised task was successful in reducing side biases. Origin had no effect on perseveration of the birds' responses (*U* = 29, *N*_wc_ = *N*_hr_ = 7, *P* = 0.620; Fig. 4c). The low *P* values of the chi-square test indicate a relatively high perseveration level in all birds. Perseveration was not correlated with measures of general activity, use of cage walls or our route-tracing metrics (*r*_S_ < 0.464, *N*_total_ = 14, *P* ≥ 0.150 in all cases).

### Risk Sensitivity in Delay to Reward

**Figure 5:**
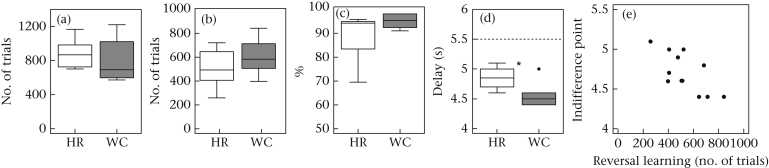
Performance in the risk sensitivity task. (a) Discrimination learning, (b) reversal learning, (c) preference of the reward associated with a variable delay as compared to a fixed 10 s delay reward, (d) indifference point after 5 days of titration. (e) correlation between indifference point and reversal learning. Dashed line in (d): harmonic mean of 3 and 15 s. Asterisk indicates significant difference (*P* ≤  0.05). Box plots as explained in legend of Fig. 3. HR: hand-reared; WC: wild-caught.

Origin had no effect on discrimination learning (step 1: *U* = 31, *N*_wc_ = 6, *N*_hr_ = 8, *P* = 0.414; Fig. 5a), on the subsequent reversal learning (step 2: *U* = 31, *N*_wc_ = 6, *N*_hr_ = 8, *P* = 0.414; Fig. 5b), or on the initial strength of preference for the VD stimulus (*U* = 33, *N*_wc_ = 6, *N*_hr_ = 8, *P* = 0.282; Fig. 5c).

Wild-caught birds had lower indifference values than the hand-reared birds (*U* = 31.5, *N*_wc_ = 6, *N*_hr_ = 6, *P* = 0.026; Fig. 5d) indicating greater impulsivity. The indifference values were weakly related to learning during the process of titration: birds with low indifference values took fewer trials during step 1 of discrimination learning (acquiring a preference for the variable delay when the fixed delay was set to 10 s) than birds with higher indifference values (*r*_S_ = 0.512, *N*_total_ = 12, *P* = 0.089), but they took more trials during step 2 (reversal learning) to change their preference from the variable to the fixed delay when the fixed-delay value was changed from 10 s to 3 s (*r*_S_ = −0.575, *N*_total_ = 12, *P* = 0.050; Fig. 5e). These correlations strengthen the validity of the task: the greater the distance between the indifference point of an individual bird and the presented FD value, the stronger the preference, that results in the bird reaching the criterion within fewer trials.

The indifference values were weakly negatively correlated with general activity (*r*_S_ = −0.560, *N*_total_ = 12, *P* = 0.058), and significantly negatively correlated to T-pattern occurrences (*r*_S_ = −0.610, *N*_total_ = 12, *P* = 0.035), but not with use of cage walls or number of different T patterns (*r*_S_ < 0.406, *N*_total_ = 12, *P* ≥ 0.201).

### Interval Timing

The fitted polynomial functions yielded adjusted *R*^*2*^ values of 0.857 on average (range 0.688–0.961). No measure of timing performance (accuracy, precision or motivation) was affected by origin (*U* < 40, *N*_wc_ = 7, *N*_hr_ = 8, *P* ≥ 0.385). Furthermore, there was no correlation between timing performance and any of our movement metrics (*r*_S_ *<* 0.446, *N*_total_ = 15, *P* ≥ 0.130).

## Discussion

Using a battery of neuropsychological tasks we compared the cognitive performance of hand-reared and wild-caught starlings. Our primary aim was to test the hypothesis that hand rearing induces impairments in basic cognition coupled with increased anxiety and impulsivity. Our results show very little evidence for any cognitive impairment in the hand-reared birds. We found no evidence that hand-reared birds were impaired in associative learning or interval timing and no evidence that they suffered from more perseverative cognition. Contrary to our predictions, we found that hand-reared birds were less neophobic in the novel stimulus task and less impulsive on the risky decision-making task than wild-caught birds. Our secondary aim was to compare the cognition of birds with and without behavioural stereotypies, such as somersaulting and route tracing, and test the hypothesis that more stereotypic starlings have more perseverative cognition. However, we found no evidence that more stereotypic birds were more perseverative, as measured by an extinction task and a two-choice guessing task. We discuss these findings in the next sections.

Contrary to our predictions, the results from the autoshaping task suggest that, if anything, hand-reared birds apparently learnt faster than wild-caught birds: hand-reared birds tended to start pecking after fewer trials, and pecked faster within individual trials. However, these results should be interpreted with caution. While it is possible that the behavioural differences recorded reflect an underlying difference in the speed with which the birds acquired an association between the stimulus (illuminated pecking key) and reward (food), it is also possible that the differences reflect a difference in neophobia towards a stimulus not previously encountered (the illuminated pecking key used as the conditioned stimulus). The results from our novel stimulus task, in which the illuminated pecking key changed colour, show that hand-reared birds were faster to peck the novel-coloured key; we therefore suggest that the most parsimonious interpretation of the autoshaping result is that the hand-reared birds were similarly less neophobic the first time they saw the stimulus in the autoshaping task. Further support for this hypothesis comes from our finding that the number of trials taken to initiate a key peck during autoshaping was positively correlated with the number of trials taken to initiate a key peck during the novel stimulus task. We therefore interpret the speed of task acquisition during the autoshaping procedure as a measure of neophobia as opposed to underlying cognitive ability. In support of this conclusion, other measures of speed of learning obtained from the risk sensitivity in the delay to reward task (trials to initial discrimination learning in step 1 and trials to reversal in step 2), which are not confounded with neophobia, show no difference between the hand-reared and wild-caught birds. Finally, it is worth noting that although previous studies have suggested a link between early life stress and impaired associative learning in other passerine bird species (Fisher et al. 2006; Bonaparte et al. 2011), it is possible that stimulus novelty could be a similar confound in these studies, and that faster learning might be attributable to lower neophobia levels. We conclude that we have no evidence to support a difference in speed of learning between the hand-reared and wild-caught birds. However, there is evidence to suggest that the hand-reared birds are less neophobic.

Seemingly at odds with the above result, in a previous study designed to compare neophobia in hand-reared and wild-caught starlings we found no difference between the groups in the latency to approach a novel object (Feenders et al. 2011). However, the tests used in this previous study were very different from those used in the current study. In the previous study, the birds were free either to approach or to ignore a novel object placed in the cage, whereas in the current study, they had strong motivation to approach the novel stimulus because they were food deprived and pecking the stimulus was associated with obtaining faster food reward. Thus, it is possible that the current tests provided a more sensitive measure of neophobia, because motivation to obtain food was placed in direct conflict with fear of the illuminated pecking key. Importantly, there were no differences in body condition between the hand-reared and wild-caught birds in the current study, suggesting that the differences in neophobia were unlikely to be driven by differences in hunger. Our finding in the current study that hand-reared birds are less neophobic fits well with other previous results from starlings showing that they are also less fearful of humans than wild-caught birds (Feenders & Bateson 2011; Jayne et al. 2013).

The second major difference between the hand-reared and wild-caught birds to emerge from the current study is that the hand-reared birds displayed evidence of less impulsive decision making. To measure impulsivity we presented the birds with a choice between a fixed delay to reward and a risky delay to reward of equal mean delay that was either shorter or longer with equal probability and titrated the value of the fixed delay until the birds were indifferent between the two options. We found that the hand-reared birds had indifference values at approximately the harmonic mean of the two delays in the risky option, as expected based on previous research (Bateson & Kacelnik 1996), but that the wild-caught birds had indifference values that were significantly lower. A lower indifference value indicates steeper discounting of delayed rewards, and hence more impulsive choice (Bateson & Kacelnik 1997; Kacelnik & Bateson 1997).

Our finding of both lower neophobia and lower impulsivity in the hand-reared birds is in direct contrast to our predictions based on our review of the known effects of early life stress and maternal deprivation in mammals. For example, monkeys that have experienced various forms of early life stress including maternal separation or variable food provision show increased incidence of anxiety and impulsivity as adults (Stevens et al. 2009). Taken together, our results suggest that hand rearing does alter emotionally mediated decision making in birds, but in a direction usually associated with lower levels of developmental stress in mammals. Although the hand-reared birds were removed from their parents at an earlier age, it is possible that parental deprivation per se is less important in birds than mammals, and that our hand-reared birds may actually have had a less stressful early life than the wild-caught birds, perhaps because they faced no competition for food during hand rearing (chicks were fed to satiation during hand rearing). However, we should be cautious in our conclusions at this stage, because a connection between early life stress and increased neophobia has not been established in birds. Indeed, male zebra finches fed corticosterone as chicks to mimic the hormonal effects of early life stress actually showed reduced neophobia in a novel object test when tested as independent juveniles (Spencer & Verhulst 2007). These results suggest either that the response to early life stress is different in birds and mammals or that novel object tests are a poor measure of anxiety/fearfulness. Further data on stress physiology in starlings will be necessary to understand more fully the suite of changes present in the hand-reared birds and to test the possibility that hand-reared chicks may have experienced lower stress levels than those raised by their parents in the wild.

We found no difference in cognitive performance between hand-reared and wild-caught birds in our measures of either perseverative cognition or interval timing performance. The development of perseverative cognition is strongly linked with various forms of developmental stress in both humans and animals (e.g. Powell et al. 1999; Garner et al. 2003b; Jones et al. 2011; Pomerantz et al. 2012), and interval timing is also an extremely sensitive measure of normal neural function (Paule et al. 1999; Matell & Meck 2004). Therefore, we are confident in asserting that based on our evidence, hand rearing, at least when following our procedures, was not a major source of developmental stress and did not result in pathological changes in the basal ganglia circuits involved in behavioural inhibition and interval timing.

The prediction that more stereotypic individuals would show evidence of more perseverative cognition was a major motivator for this project. We began this project with the hypothesis that hand-reared starlings would be more stereotypic, and that birds with stereotypies would be more perseverative, resulting in prolonged pecking in the extinction task, greater sequential dependency in the two-choice guessing task and less accurate and less precise interval timing functions (Garner et al. 2003a, b). However, our predictions failed on two levels. First, we found no evidence that hand-reared starlings were more stereotypic than wild-caught birds (cf. Mason 2006). On the contrary, in a previous paper in which we described the development of stereotypies in the same group of birds used in the current study, we reported that somersaulting stereotypies only developed in the wild-caught birds, and that route-tracing stereotypies were also potentially more severe in the wild-caught birds (Feenders & Bateson 2012). Second, in the current study we found no evidence that more stereotypic starlings had more perseverative cognition. In our wild-caught group of birds those birds that developed elements of the somersaulting stereotypy performed similarly to the normal birds on the extinction task. Similarly, we found no association between our metrics of route tracing and any of our measures of perseverative cognition derived from the extinction task, the two-choice guessing task or the interval timing task.

We discuss three alternative explanations for our negative findings (see also Gross et al. 2011 for similar arguments). First, it is possible that the tasks we used did not adequately probe for perseveration (more specifically, recurrent perseveration, see Sandson & Albert 1984), perhaps because the intertrial interval in the two-choice guessing task in part A was relatively long at 150 s meaning that the birds could move away from the pecking panel and hence break a perseverative sequence of behaviour. To address this possibility, the two-choice guessing task in part B was set up to be more similar to the test previously used successfully to show a correlation between stereotypy and cognitive perseveration in parrots (Garner et al. 2003b). However, even with this modified test we did not find any correlation, suggesting that the negative result we obtained was not explained by our choice of tasks. Second, it is possible that the stereotypic behaviour that we observed in the starlings was not associated with pathological behavioural disinhibition. A recent study of laboratory mice, *Mus musculus*, also found no correlation between cage stereotypies and cognitive perseveration (Gross et al. 2011). The latter authors measured a number of different stereotypic behaviour patterns and argued that perhaps only a subset of these were associated with perseveration. In support of this hypothesis, in deer mice, *Peromyscus maniculatus*, only animals with complex somersaulting stereotypies performed more poorly in a reversal task whereas mice exhibiting other simpler jumping stereotypies did not (Tanimura et al. 2008). Thus, perhaps only some complex abnormal stereotypic behaviour patterns are associated with cognitive inflexibility. It is possible that we tested our birds at a time point when cage-induced stereotypies were only starting to develop and the birds' basal ganglia circuits were still functioning normally. A study with tits that reported perseverative cognition associated with route-tracing stereotypies used birds that had been caged for 3 years (Garner et al. 2003a), which is much longer than the maximum continuous period spent in cages by the starlings in the current experiment (36 days). Finally, it is possible that the link between stereotypies and perseverative cognition may not be as tight or as simple as previously thought. In a recent study on monkeys, perseveration in a reversal learning task correlated with self-directed behaviour but not with other behaviour categories such as locomotion (Judge et al. 2011). The authors did not observe any stereotypies and argued that all the recorded behaviour was in the normative range. Thus, cognitive perseveration can be related to motor patterns that are not obviously abnormal. The important conclusion to emerge from this part of our research is that even in starlings with measurable somersaulting and route-tracing stereotypies, we were unable to detect any evidence for perseverative cognition indicative of pathological changes in the basal ganglia circuits. Although our birds were individually caged in relatively small, barren cages and thus potentially at risk of developing stereotypies (Asher et al. 2009), it is perhaps significant that the birds were never caged for a continuous period of more than 40 days without being returned to the aviary for a break. It is possible that this regime prevented the development of more severe, crystallized stereotypies associated with basal ganglia pathology.

As a final point, it is worth noting that the use of hand-reared birds in behavioural research is likely to become more widespread in the near future. Recent changes in European Union legislation (Directive 86/609/EEC, revision 2010/63/EU) place restrictions on the use of wild animals in scientific procedures (Council of the EU 2010). Where wild species must be used and captive breeding is not possible, hand rearing of very young animals taken from the wild is being promoted (e.g. by the U.K. Home Office) as a strategy that will address the welfare objective of the law. We have been concerned that there has been insufficient attention paid to the potential welfare and scientific problems associated with a widespread switch to the use of hand-reared birds (Feenders & Bateson 2011; Feenders et al. 2011; Jayne et al. 2013). Wild bird species are generally used in research because they offer natural behavioural traits not found in typical laboratory species (Bateson & Feenders 2010), and if their behavioural development were compromised by hand rearing this would reduce the value of such research. The starling is the most widely used wild passerine species in laboratory research, and to date the vast majority of starlings used in research have been captured from the wild as independent juveniles or adults (Asher & Bateson 2008; Bateson & Feenders 2010). Our study is therefore important in demonstrating that the behavioural development of the European starling does not appear to be compromised by hand rearing. Indeed, if anything, our findings suggest that the welfare of hand-reared starlings in the laboratory is better than that of wild-caught birds owing to reduced incidence of stereotypic behaviour and reduced fearfulness.

It is difficult to prove negative results conclusively with a relatively small sample of birds. However, our sample size of 32 starlings was larger than that used in typical cognitive studies of passerine birds. For example, the four studies of corvids referred to in the introduction used a maximum of 23 subjects (Clayton & Dickinson 1998: 23 birds; Seed et al. 2007: 10 birds; Hoffmann et al. 2011: six birds; Schwab et al. 2012: 12 birds). Therefore, we can be confident in concluding that even if hand rearing does result in some cognitive impairments in starlings, then these impairments are small effects compared with other cognitive phenomena studied for which significant findings can be obtained with smaller numbers of birds. It is also worth noting that we did not study all aspects of cognition in our birds, and it is possible that hand rearing produced deficits in domains that we did not measure such as spatial or social cognition. Of note, a comparison of spatial cognition in wild-caught and hand-reared black-capped chickadees, *Poecile atricapillus*, found no evidence for effects of rearing conditions on spatial cognition (Batty et al. 2009).

### Conclusions

Our neuropsychological tests indicate that hand rearing does not affect basic cognitive performance in starlings. Additionally, we found no evidence that birds that developed stereotypies in laboratory cages (predominantly wild-caught birds in this study) had any cognitive impairments, although this may be because none of our birds developed severe, crystallized stereotypies. In contrast, we found some evidence that emotionally driven decision making was altered in a direction usually associated with reduced developmental stress. This study therefore supports the use of hand rearing as an experimental procedure in behavioural research on starlings, at least when periods in small cages are frequently interspersed by social housing in large aviaries. Further research will be required to establish whether this finding generalizes to other husbandry regimes and other passerine species.
